# Commentary: Hand and Grasp Selection in a Preferential Reaching Task: The Effects of Object Location, Orientation, and Task Intention

**DOI:** 10.3389/fpsyg.2016.01129

**Published:** 2016-07-28

**Authors:** Quentin Moreau, Matteo Candidi

**Affiliations:** ^1^Social and Cognitive Neuroscience Laboratory, Department of Psychology, Sapienza University of RomeRome, Italy; ^2^Istituto di Ricovero e Cura a Carattere Scientifico, Fondazione Santa LuciaRome, Italy

**Keywords:** joint-action, grasping kinematics, social grasping, task intention, reach-to-grasp

Reach-to-grasp actions are the outcomes of several voluntary sub-movements (Jeannerod, 1981, 1984; Paulignan et al., 1997) dependent on the object intrinsic position and shape characteristics (Fattori et al., 2009). The behaviors implemented in order to achieve the proximal goal (i.e., grasping) are further modulated by the action one wants to perform once the object has been grasped (distal goal; Cohen and Rosenbaum, 2004; Cattaneo et al., 2007). These findings suggest that the reason why an object is grasped has an effect on initial prehension kinematics (i.e., “end-goal effect”).

Grasping an object often serve the purpose of interpersonal interactions, which are defined by matching behavioral adaptation between subjects (Baldwin, 1992) and, more importantly, joint actions, where the presence of a common goal bounds individuals' behaviors (Sebanz et al., 2006). In these interactive scenarios, the intentions one attributes to his partner are crucial in shaping his own hand-preference for grasping objects as well as the kinematics of his movements. For these different features to be integrated in one movement, reach-to-grasp has been used as a paradigmatic example of the interface between motor behavior and cognition.

Recently, Scharoun et al. (2016) aimed at studying hand-preference in an ecological set-up including individual and so-called “joint actions” conditions. Here, we argue that adding a social aspect to an individual condition does not capture the essence of joint actions. The authors asked subjects to reach-and-grasp a mug in order to perform four different actions without giving them any instructions on how to perform their grasping. Such paradigms contribute to the field of motor control by clarifying grasping hand-preference embedded in the strategy of grasping. Furthermore, Scharoun and her colleagues proposed four different experimental conditions to test the role of the distal goal on hand-preferences: (1) pick-up (unimanual, independent); (2) pick-up and pour (bimanual, independent); (3) pick-up and pass (unimanual, joint action); and (4) pick-up, pour, and pass (bimanual, joint action). The behavioral results of the study show that the first two conditions offer clear support for the dominant hand preference hypothesis during unimanual actions. The study needs to be praised for showing all the aforementioned effects in a realistic paradigm.

However, we think that calling “joint actions” their third and fourth conditions is misleading and represent a misuse of the “joint action” terminology and its actual conceptualization. Indeed, to their “independent” condition, Scharoun and colleagues chose to oppose the term “joint action” when participants had to pass the mug to a confederate who was simply sitting in front of them.

We contend that joint actions are characterized by specific features and need to be called into play with caution. One crucial feature of joint actions is that they are activities involving two or more individuals who need to voluntarily coordinate their actions in time and space in order to achieve a desired change in the environment (Sebanz et al., 2006). In Scharoun's study, we argue that their set-up is not in phase with this definition, making their “joint action” condition contestable and more similar to a “social condition.” We do not deny the interest of studying the way in which objects are grasped *before* passing them to another person (Becchio et al., 2008b) which is clearly inherent to social collaboration.

By choosing a passive experimenter as a confederate, Scharoun and colleagues' set-up fails to measure the dynamic encounters that adjust behavioral and cognitive processes of agents involved in joint actions (Knoblich and Sebanz, 2006; Sebanz et al., 2006; Vesper et al., 2010; Di Paolo and De Jaegher, 2012; Dolk et al., 2014; Sacheli et al., 2015b). Even though the “pick up and pass” and the “pick-up, pour and pass” conditions show a different hand-preference pattern compared to the individual ones, these modulations may be a consequence of the confederate mere presence (i.e., social affordance; Becchio et al., 2008b; Ferri et al., 2011). Therefore, by lacking a condition directly testing the role of a social request (Ferri et al., 2011) to identify the direct influence of the possible *interaction* with a confederate, these modulations should not be interpreted as a result of a joint action.

Since humans and primates share grasping behaviors, a great number of monkeys and humans studies have been using reach-to-grasp actions which gained us a good understanding of grasping physiology (Castiello, 2005). For these reasons, grasping has also been used in “social contexts” (Becchio et al., 2008a, 2012; Rozzi and Coudé, 2015) during interpersonal interactions or, crucially, adopting joint actions paradigms. Recording kinematics and/or brain dynamics during these interactions improved the knowledge of social neurosciences in non-verbal communication, coordination, competition, and leader–follower situations (Rizzolatti and Fadiga, 1998; Ménoret et al., 2014; Candidi et al., 2015a; Sacheli et al., 2015a,b).

After years of philosophical and scientific debates, social neurosciences need to now focus on “online,” dynamically mutual, motor interactions (Schilbach et al., 2013), where “individualism” steps aside for “interactionism” to rise (Gallotti and Frith, 2013). This suggests creating experimental paradigms that allow partners' reciprocal and bidirectional adjustments during the interaction (Sacheli et al., 2015a). Such online paradigms allow closed-loop processes (Hari and Kujala, 2009; Tognoli and Kelso, 2015) that bound together individuals and constrain their individual behavior (Candidi et al., 2015b). Experimentally speaking, this requires bidirectional set-ups, where contributors' motor action and perception allow a shared representation of the action between all participants (Sebanz et al., 2003, 2005, 2007).

In conclusion, we agree with Scharoun and her colleagues' claims on the importance of creating ecological set-ups. But in the field of joint actions these should be developed with the constant constraint of involving reciprocal and bidirectional adaptation between two agents. Thus, we argue that Scharoun and colleagues' “non”-independent condition was not a joint action *per se*.

In order to bring the field of joint action to the forefront of social neurosciences and build credibility concerning the related literature, one should beware of their essential features and use the joint action terminology prudently and parsimoniously. This is a fundamental condition to attain greater continuity and coherence in our field.

## Authors contributions

QM and MC have made substantial, direct, and intellectual contribution to the work, and approved it for publication.

### Conflict of interest statement

The authors declare that the research was conducted in the absence of any commercial or financial relationships that could be construed as a potential conflict of interest.
